# Effects of intrinsic and extrinsic growth factors on virulence gene expression of foodborne pathogens in vitro and in food model systems; a review

**DOI:** 10.1002/fsn3.4281

**Published:** 2024-06-19

**Authors:** Hedayat Hosseini, Razzagh Mahmoudi, Babak Pakbin, Leila Manafi, Setayesh Hosseini, Zahra Pilevar, Wolfram Manuel Brück

**Affiliations:** ^1^ Food Sciences & Technology Department, National Nutrition & Food Technology Research Institute, Faculty of Nutrition & Food Technology Shahid Beheshti University of Medical Sciences Tehran Iran; ^2^ Medical Microbiology Research Center Qazvin University of Medical Sciences Qazvin Iran; ^3^ Health Products Safety Research Center Qazvin University of Medical Sciences Qazvin Iran; ^4^ Institute for Life Technologies University of Applied Sciences Western Switzerland Valais‐Wallis Sion Switzerland; ^5^ Department of Cell and Molecular Biology Sciences, School of Biology, College of Science University of Tehran Tehran Iran; ^6^ School of Health Arak University of Medical Sciences Arak Iran

**Keywords:** gene expression, growth factors, stress condition, virulence genes

## Abstract

Since foodborne diseases are one of the major causes of human hospitalization and death, one of the main challenges to food safety is the elimination or reduction of pathogens from food products throughout the food production chain. Pathogens, such as *Salmonella* species, *Escherichia coli*, *Bacillus cereus*, *Clostridium* species, *Staphylococcus aureus*, *Listeria monocytogenes*, *Campylobacter* species, etc., enter the consumer's body through the consumption of contaminated food and eventually cause disease, disability, and death in humans. In particular, the expression of virulence genes of these pathogens in various food environments containing them has been repeatedly reported, which is a key issue for the survival and pathogenicity of the pathogen. Hence, in this review, the interventions to prevent and control foodborne diseases, such as the application of natural preservatives, redox potential, heat treatments, high‐pressure processing, and gaseous atmosphere, are discussed based on the literature. Moreover, the effects of various environmental conditions on bacterial gene expression are comprehensively reviewed. In conclusion, the effects of intrinsic and extrinsic factors on the growth and pathogenicity of bacteria are very complicated. The information obtained from the current study can be used to develop new control strategies, improve food safety, and ensure human health.

## Graphical abstract

Since foodborne diseases are one of the major causes of human hospitalization and death, one of the main challenges to food safety is the elimination or reduction of pathogens from food products throughout the food production chain. In this review, the interventions to prevent and control foodborne diseases, such as the application of natural preservatives, redox potential, heat treatments, high‐pressure processing, and gaseous atmosphere, are discussed based on the literature. Moreover, the effects of various environmental conditions on bacterial gene expression are comprehensively reviewed.

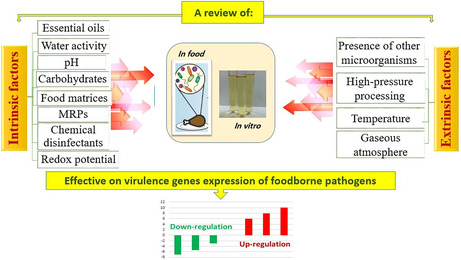

## 1 INTRODUCTION

**FIGURE 1:**
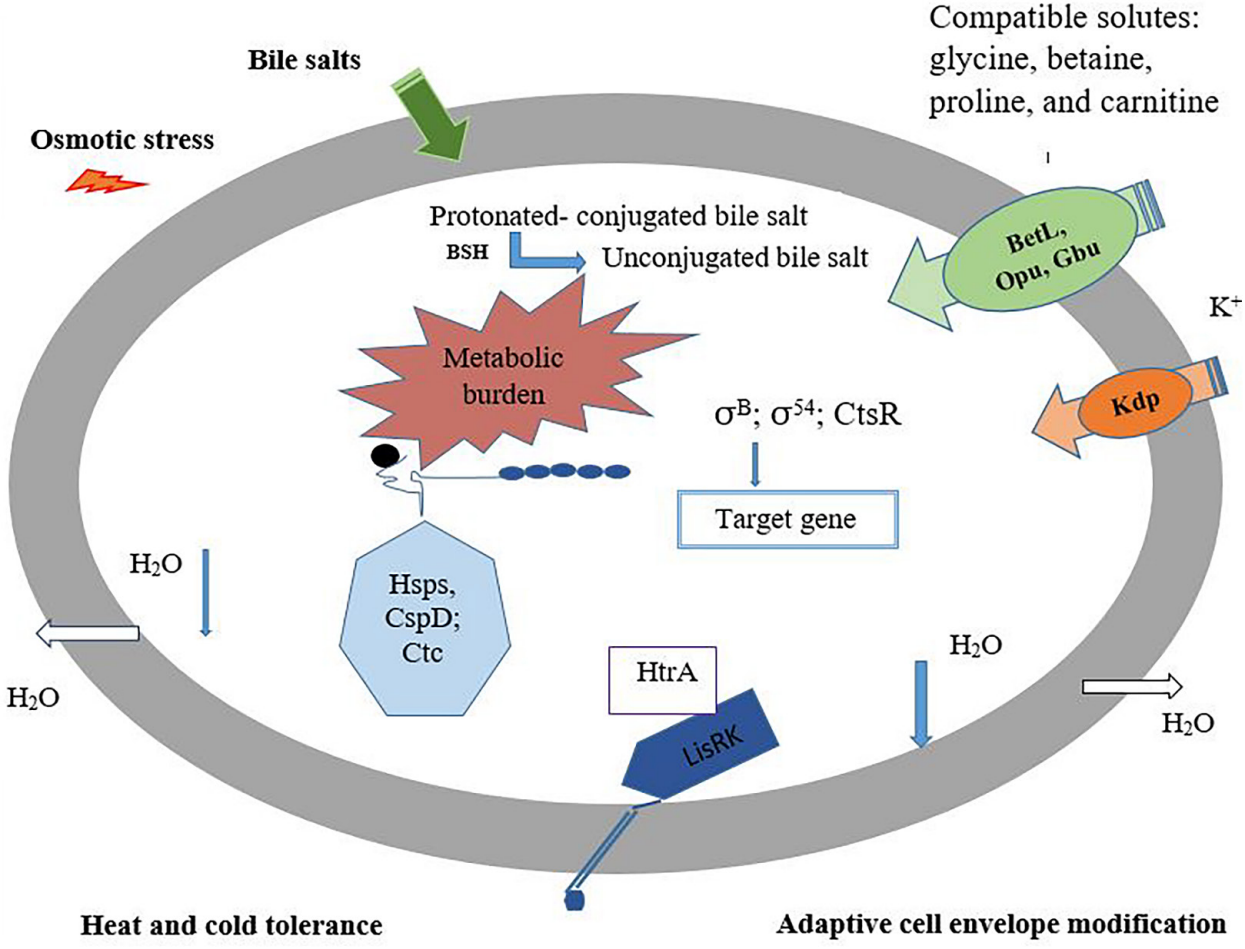
Overview of the adaptation systems of *L. monocytogenes* to cold, heat, bile, and osmotic stress. At high osmolarity conditions, K^+^ initially enters the cell then compatible solutes such as betaine, glycine, proline, and carnitine. Under cold stress, these solutes also accumulate inside the cell. In both osmotic and cold stress, cold shock proteins (Csps) play an important role in the tolerance response. CstR regulators are involved in the tolerance response under osmotic conditions and heat shock. In the absence of protective osmolytes in the cell, Ctc participates in the osmotolerance response. LisRK two‐component regulatory system involved both osmoregulatory and osmosensing functions. Protonated‐conjugated bile salts under the influence of BSH break down into non‐conjugated bile salts in the bile stress (Faleiro, 2017).

**FIGURE 2:**
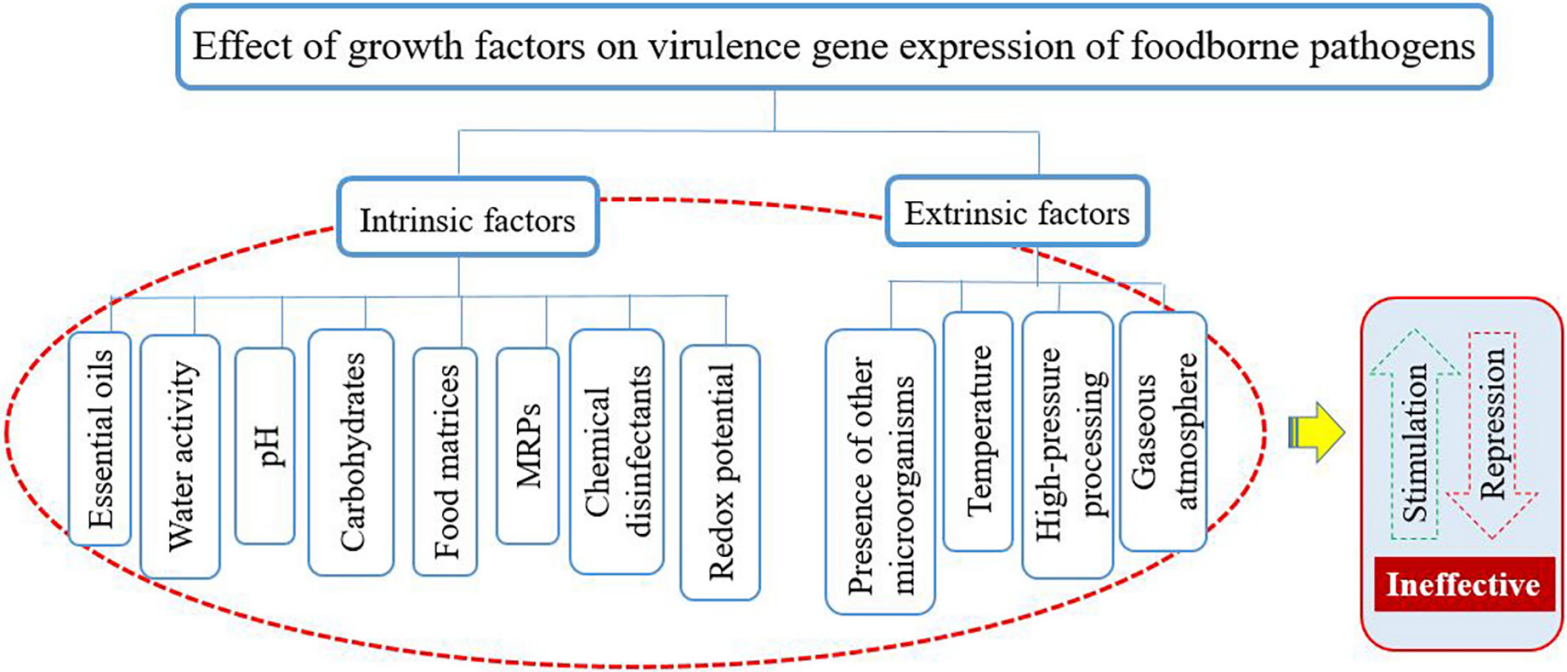
A schematic review of the factors affecting the expression of virulence genes of foodborne pathogens.

Currently, achieving safe and adequate food and thus ensuring consumers’ health is one of the major concerns around the world (Hosseini et al., 2004; Msimango et al., 2023; Wang et al., 2021). Based on the reports, food products contaminated with foodborne pathogens, such as *Salmonella* species, *Escherichia coli*, *Bacillus cereus*, *Clostridium* species, *Staphylococcus aureus*, *Listeria monocytogenes*, *Campylobacter* species, etc., are among the major causes of death in developing countries (Larsen & Jespersen, 2015; Manafi et al., 2023; Mohammadi et al., 2022; Mousavinafchi et al., 2023). In the recent decade, nearly 420,000 case deaths, 600 million case diseases, and 33 million years of potential life lost due to foodborne diseases have been reported worldwide (Bakshi et al., 2024; Pires & Devleesschauwer, 2021). Indeed, the pathogens enter the food during the production, packaging, transportation, and distribution stages (in general, different stages of the farm‐to‐fork) making them more difficult to control (Cálix‐Lara et al., 2014; Manafi et al., 2020; Somda et al., 2018). Foods are rich in nutrients for bacteria to grow, however, bacteria are exposed to many stressful conditions (such as limited nutrient accessibility, osmolarity, inappropriate pH, oxidation, excessive temperatures, and extreme physical and chemical stresses) in the food environment during processing and storage (Alvarez‐Ordóñez et al., 2015; Roy et al., 2021). Moreover, it has been proven that some cells adapt quickly to different conditions, overcome stresses, and grow under the influence of internal (such as proteins, carbohydrates, fats, vitamins, additives, and nutrients) or external factors (temperature, storage time, etc.) of the food environment, and eventually cause disease in the consumer (Alvarez‐Ordóñez et al., 2015; Faleiro, 2017). For example, Figure 1 illustrates an overview of the adaptation systems of *L. monocytogenes* to some stress conditions. In general, following bacterial cells' exposure to stress conditions, to maintain viability and bacterial compatibility, changes occur in the gene expression pattern of the bacteria and subsequently affect virulence potential (Alvarez‐Ordóñez et al., 2015). More accurately, bacteria use these environmental signals to better sense the environment and regulate gene expression accordingly (Villoria Recio et al., 2020). Searches show that virulence genes of foodborne pathogens in various food environments have been significantly expressed, which is considered a key process for the survival and pathogenicity of the pathogen (Larsen & Jespersen, 2015). Therefore, investigators use different chemical and natural compounds to control and suppress these microorganisms in foods and due to the increasing awareness of consumers about the possible dangers of chemical additives, the use of natural additives has become more common (Aguilar‐Veloz et al., 2020; Farhanghi et al., 2022; Ghalehnovi et al., 2023; Saatloo et al., 2023; Yazdanfar et al., 2023). Although extensive studies have been conducted to control the growth of foodborne pathogens, relatively little and scattered information has been published on how to control and regulate the virulence gene expression of these pathogens in food. Hence, the current study provides a comprehensive review of various compounds and methods used to regulate the virulence gene expression of foodborne pathogens in vitro and in food models (Figure 2).

In the following, the effect of intrinsic growth factors, such as essential oils, water activity, pH, carbohydrates, Maillard reaction products (MRPs), food matrices, and redox potential, and extrinsic growth factors, such as the presence of other microorganisms, temperature, high‐pressure processing (HPP), chemical disinfectant, and gaseous atmosphere on virulence gene expression of foodborne pathogens, has been described in detail. Some of the key virulence genes of foodborne pathogens are listed in Table 1.

**TABLE 1:** A list of the most key virulence genes of foodborne pathogens.

Foodborne pathogens	Virulence gene of bacteria	References
*L. monocytogenes*	*prfA, plcA, plcB, Hly, actA, Iap, intA, intB, motA, and motB*	Schiavano et al. (2022), Upadhyay et al. (2012)
*E. coli* O157:H7	*fliA, fliC, motA, cheA, cheZ, sepD, escC, stx2A, stx2B, stx1A, stx2A*	Allen and Griffiths (2012), Mei et al. (2017)
*S. enteritidis*	*invH, rpoS, sipA, sipB, sodC, spvB, mgtC, sopB*	Upadhyay et al. (2017)
*S. typhimurium*	*hilA, invA, sopB, mgtC*	El‐Azzouny et al. (2018), Giovagnoni et al. (2020)
*S. aureus*	*spa, sea, sae, tst, agrA, sbi*	Ramezani et al. (2019)
*C. jejuni*	*cadF, cdtB, ciaB, flaA*	Mundi et al. (2013)
*B. cereus*	*nheA, nheB, nheC*	Yu et al. (2019)

## 2 INTRINSIC AND EXTRINSIC FACTORS AFFECTING GENE EXPRESSION OF FOODBORNE PATHOGENS

### 2.1 Intrinsic factors

#### 2.1.1 Essential oils and extracts

Nowadays, the use of plant‐derived essential oils in food preservation has remarkably increased (György et al., 2020; Hosseini Khabbazi et al., 2023; Pilevar et al., 2017; Wu et al., 2015). So far, many studies have been performed on the effect of essential oils on regulating virulence gene expression in foodborne bacteria, according to them, essential oil compounds inhibit foodborne bacteria growth and virulence gene expression (Ben Akacha et al., 2024; Frazzon et al., 2018; Huang et al., 2023; Zou et al., 2020). For example, the expression of virulence genes including *prfA*, *fur*, *hly*, *actA*, and *agrA* was downregulated in *L. monocytogenes* by exposure to essential oil extracted from the *Baccharis psiadioides* plant. This function is probably related to a high concentration of a monoterpene called β‐pinene, which is known as the dominant antimicrobial compound in several plant‐derived essential oils (Pieta et al., 2017). Besides, the expression of *S. typhimurium* virulence genes, including the type III secretion system (*T3SS*) and type I fimbriae (*TIF*) genes, was significantly down‐regulated when exposed to sub‐minimal inhibitory concentration (sub‐MIC) of eugenol in vitro (Zhao et al., 2022). While the transcription of *hilC*, *hilD*, *sicA*, and *sprB* genes was down‐regulated in *S. enteritidis* phage type 8 (PT8) exposed to both trans‐cinnamaldehyde and eugenol, the induction of *SipA*, *SipB*, *SipC*, *SipD*, and *sopB* was solely down‐regulated by trans‐cinnamaldehyde (Kollanoor Johny et al., 2017). Real‐time polymerase chain reaction (PCR) data from another study also revealed that exposure to 0.005% mL. g^−^ of *Carum copticum* essential oil up‐regulated *stx* gene expression in *E*. *coli* O157:H7 at all of the assessment times (0, 24, 48, and 72 h) in 15°C Trypticase soy broth (TSB) media; in contrast, the tendency to increase by exposure to higher concentrations (0.015%) was observed only in the first 24 h (Mahmoudzadeh et al., 2020), and interestingly, a considerable down‐regulation was observed in the expression of this gene in inoculated *E*. Coli O157:H7 into minced beef, in treatment with concentrations above 0.5% of essential oil, at 4°C (Mahmoudzadeh, Hosseini, Hedayati, et al., 2016). Overall, this down‐regulation of the gene expression was attributed to the lower storage temperature, essential oil concentrations, higher iron content, and inhibitory compounds to suppress autoinducer‐2 (AI‐2) activity in minced meat compared to the broth medium (Mahmoudzadeh, Hosseini, Hedayati, et al., 2016). Especially, it was previously proven that the expression of virulence genes of *E. coli* O157:H7 (*yadK* and *hhA*) was up‐regulated by AI‐2 only, but in the ground beef extract containing AI‐2, the induction effect of AI‐2 on the gene expression is inhibited and the gene expression is suppressed (Soni et al., 2008). In addition, an up‐regulation of gene expression was observed in *L. monocytogenes* when exposed to *Zataria multiflora* Boiss. essential oil in minced fish and broth media. The gene expression regulation exhibited a mixed response at various essential oil concentrations, incubation times, and temperatures (Hosseini et al., 2021). Therefore, the growth and virulence potential of bacteria in different conditions of temperature and incubation time were affected by concentrations of essential oils (Pilevar, Hosseini, Abdollahzadeh, et al., 2020). On the other hand, Upadhyay et al. (2017) described that the effect of subinhibitory concentrations of essential oils on the regulation of *Campylobacter jejuni* pathogenesis gene expression in various strains is different (Upadhyay et al., 2017). Moreover, the transcription of *ler, stx2B, flci, and luxS* virulence genes of *E. coli* O157: H7 was down‐regulated in a concentration‐dependent manner by both oregano (*Origanum heracleoticum*) and carvacrol essential oils (Mith et al., 2015). It has also been reported that *E. coli* pathogenic gene expression regulation, such as *stx2*, *aggR*, *pic*, and *rpoS*, in response to plant compounds did not follow a compatibility pattern and were compound‐ and strain‐dependent in vitro (Garcia‐Heredia et al., 2016). Evaluation of the effect of lemongrass essential oil on the expression of virulence genes of different strains of *L. monocytogenes* also revealed that the *hly* and *inlJ* gene expression was down‐regulated in all strains of *L. monocytogenes* and the down‐regulating effect became more severe with increasing the essential oil concentration. On the other hand, *plcA*, *plcB*, *inlB*, *inlC*, and *lmo2470* genes were down‐regulated in a dependent manner on the *L. monocytogenes* strains, but no regulation effects of *prfA*, *inlA*, *sigB*, and *lmo2672* genes were reported (Hadjilouka et al., 2017). Furthermore, researchers have confirmed varying degrees of moringa oil's inhibitory effect on the expression of *L. monocytogenes* virulence genes including *prfA*, *plcA*, *hly*, *actA*, *plcB*, *inlA*, and *inlB*. Moringa oil as a natural preservative contains various antimicrobial compounds including palmitic acid, stearic acid, and oleic acid (Cui et al., 2020).

Likewise, the evaluation of the efficiency of thymol nanoemulsion inhibiting antibiotic‐resistant foodborne pathogens exhibited a down‐regulation in the *sopB* virulence gene expression after exposure to various concentrations of nanoemulsion in XDR (extensively drug‐resistant) *S. enteritidis* (Bendary et al., 2021). Previously, comparing the effect of free and nanoliposome forms of *Zataria multiflora* essential oil on the regulation of *E. coli* O157:H7 *stx2A* gene showed a dose‐dependent decrease in the gene regulation by both forms of essential oil, in which the down‐regulation was greater by nanoliposome form of essential oil (Khatibi et al., 2018). In this regard, encapsulation improves the biological activity of essential oils at target sites by increasing stability, modulating diffusion, and protecting them from environmental interference. Consequently, nanoliposomes are one of the most commonly approved nanocarriers by the Food and Drug Administration (FDA) used for drug delivery in hydrophilic and lipophilic types (Najafloo et al., 2020). In addition, cranberry extract and protein antimicrobial significantly down‐regulated the transcription of virulence factors in *S*. *typhimurium* (Das et al., 2019). Evaluating benzyl isothiocyanate's effects on the expression of *S*. *aureus*, *Vibrio parahaemolyticus*, and *S*. *typhimurium* virulence factors showed a down‐regulation in the virulence gene expression in the treatment group compared to the control. In general, this edible flavoring agent affects bacterial pathogenicity by potential mechanisms, such as effects on bacterial morphology, integration of biofilm structure, and bacterial membrane permeability (Niu et al., 2020; Zhang et al., 2021). Moreover, the inhibitory effect of *Melissa officinalis* essential oil on the growth and expression of *Listeria* virulence genes has recently been reported (Carvalho et al., 2023). Immersion of spinach and celery in water containing natural berry pomace extracts at low temperatures could reduce the number of *S*. *typhimurium* colonies and the regulation of key virulence gene expression depending on the treatment conditions (Alvarado‐Martinez et al., 2020). Researchers also indicated that both aqueous and alcoholic extracts of grape seeds inhibited the sea gene expression of the *S. aureus* isolated from tap water, pus, food, nasal tract, and soil. Indeed, active compounds including polyphenols, tannins, and acid in these extracts are the main reasons for their inhibitory effect (Bushra et al., 2022). The relative expression of the virulence genes of pathogens in the presence of some of the essential oils is listed in more detail in Table 2.

**TABLE 2:** The relative expression of the virulence genes of foodborne bacteria in the presence of some essential oils.

Virulence gene of bacteria		Essential oils	Relative fold change			References
*L. monocytogenes*	*prfA*	TC, CR, T	−5.64	−8.17	−12.15	Upadhyay et al. (2017)
	*plcA*		−11.76	−4.57	−6.39	
	*plcB*		−9.08	−5.71	−1.65	
	*Hly*		−14.47	−6.42	−1.13	
	*actA*		−13.41	−3.36	−13.06	
	*Iap*		−19.41	−6.44	−2.14	
	*intA*		−5.27	−17.79	−9.88	
	*intB*		−10.57	−17.61	−20.84	
	*motA*		−15.65	−4.97	−2.84	
	*motB*		−20.18	−5.84	−1.13	
*E. coli* O157:H7	*fliA*	TC, CR, T	−1.0	−5.1	−7.2	Yuan and Yuk (2019)
	*motA*		−2.7	−4.0	−2.2	
	*cheA*		−3.0	−16	−12	
	*cheZ*		−2.5	−1.8	−1.8	
	*sepD*		−2.0	−3.0	−1.0	
	*escC*		−1.0	−4.9	−3.6	
*S. enteritidis*	*invH*	CR, T, EUG	−16.39	−28.55	−18.89	Upadhyaya et al. (2013)
	*rpoS*		−4.20	−3.33	−1.33	
	*sipA*		−29.66	−78.59	−76.30	
	*sipB*		−7.12	−14.50	−50.29	
	*sodC*		−4.14	−8.67	−0.22	
	*spvB*		−0.28	−0.14	−0.12	
	*mgtC*		−4.09	−4.52	−0.12	
	*sopB*		−22.49	−43.22	−48.58	
*S. typhimurium*	*hilA*	CR, T	−4.4	−1.46		Giovagnoni et al. (2020)
	*invA*		−4.8	−1.71		
*S. aureus*	*sea*	EUG	−10.4			Qiu et al. (2010)
	*seb*		−6.3			
	*tst*		−12.8			
	*agrA*		−15.6			
	*hla*		−5.5			
*S. typhimurium*	*sopB*	TH	−0.33			El‐Azzouny et al. (2018)
	*mgtC*		−0.21			

#### 2.1.2 Water activity

Salt has long been used as a preservative in some foods. Concretely, salt improves the shelf life of foods by reducing the water activity and disrupting the electrochemical potential of microbial membrane (Hosseini et al., 2024). However, some foodborne pathogens can tolerate high osmotic stresses and retain their pathogenic properties (Faleiro, 2017). For example, transcriptional tracing of virulence‐related genes of viable but non‐culturable *E. coli O157:H7* under osmotic conditions revealed that *stx1*, *stx2*, *eae*, and *hly* genes were expressed in both treatments of distilled water and fish meat (with 10% and 30% salinity) (Khezri et al., 2020). The researchers also reported an up‐regulation of the *prfA* gene expression level in *L. monocytogenes* by prolonged exposure to osmotic and acidic conditions in vitro, due to the pathogen adaptation ability to the existing condition (Makariti et al., 2015). Another study showed that *L. monocytogenes* virulence gene expression in osmotic conditions is strain‐ and salt‐dependent. Despite the reduced growth of *L. monocytogenes* ATCC 51779 on cheese containing high and low salt concentrations, the expression of virulence genes in this strain was more affected by sodium chloride (NaCl) than of *L. monocytogenes* DSMZ 15675 strain that showed less sensitivity to salt content during growth on cheese. More accurately, the *prfA*, *actA*, *hly*, and *bsh* genes were up‐regulated in the more sensitive strain of ATCC 51779 incubated on cheese (both at low and high NaCl), however, down‐regulated or constant gene expression level was observed at all conditions on the less sensitive strain of DSMZ 15675. Furthermore, in both strains, the down‐regulation of *agrA*, *ami*, *gadC*, and *opuC* was increased on exposure to low NaCl content (Larsen & Jespersen, 2015). Bergholz et al. (2012) also showed that *L. monocytogenes* was adapted to osmotic stress in a temperature‐dependent manner so that an up‐regulation of *prfA*, *mpl*, *actA*, *plcB*, *plcA*, and *inlA* virulence gene transcription occurred in the long‐term response at 37°C, while at 7°C, proteins involved in stress response and cell modifications were regulated (Bergholz et al., 2012). Another study showed that the expression of the *prfA* gene was down‐regulated in *L. monocytogenes* grown in raw milk containing NaCl at both 25 and 37°C. However, the gene expression level at 25°C was several thousand fold lower than at 37°C. Moreover, the *prfA* gene was differently regulated in Gouda cheese prepared from raw milk artificially inoculated with *L. monocytogenes* (Carstens, 2017). The survivability of *L. monocytogenes* exposed to moderate salt and increased expression of virulence genes (*hly*, *prfA*, and *intA*) will probably lead to increased pathogenicity (Hosseini et al., 2024).

#### 2.1.3 pH

As mentioned earlier, pathogens may be exposed to lethal and sublethal concentrations of acids in the food chain, and similar to other stresses, exposure to lower concentrations leads to the pathogen adapting to the acid, which impairs the antibacterial effect of the acid in subsequent encounters and protects the bacterial cell against other stresses (such as heat, hydrogen peroxide (H_2_O_2_), and NaCl) (Faleiro, 2017). The occurrence of this reaction depends on the growth phase of the bacteria. For example, the bacteria in the stationary phase are notably compatible with acidic stress (Faleiro, 2017). Examination of the response of some *Salmonella* strains and serovars to the expression of virulence genes under acidic conditions showed that the greatest change in *hilA* gene regulation occurred within 0 and 2 h after exposure to both HCl and acetic acid. While the gene expression of *hilA* was down‐regulated or up‐regulated very little (less than onefold) in exposure to acid at time zero, it was up‐regulated after 2 h in almost all the strains. Despite having a similar overall pattern, the transcriptional level was associated with strain, serovar, and acid (González‐Gil et al., 2012). Moreover, *Stx2A* gene expression was down‐regulated by acetic acid, lactic acid, and hydrochloric acid in a concentration‐dependent manner so that the level of gene expression was lower with increasing acid concentration; meantime, acetic acid had the greatest effect on down‐regulation of gene expression compared to the other two acids (Carey et al., 2008). Rishi and Ricke inoculated *S. typhimurium* in an in vivo simulated environment containing short‐chain fatty acids (SCFAs) and indicated that the SCFAs in food preservatives and the gastrointestinal tract (GIT) were altered pH conditions (pH = 3). The *hilA* virulence gene was more expressed in SCFAs adapted cells exposed to acidic pH than in SCFAs unadapted cells exposed to acidic pH. The up‐regulation of gene expression can take place due to cellular adaptation to SCFAs and thus induce resistance in the organism, which may result from changes in the phospholipid structure of the membrane (Rishi & Ricke, 2007). In parallel, the researchers also noted that the cyclic derivatives of unsaturated fatty acids (cyclopropane fatty acids), which may be formed in cell membranes due to various stresses, are important factors in increasing *Salmonella* spp. resistance to acid stress (Alvarez‐Ordóñez et al., 2015). Moreover, an up‐regulation in expression of the *Salmonella* virulence gene (*hilA*) was also observed at acidic conditions (pH = 5) compared to neutral pH, which probably caused an increase in the virulence of the bacteria in exposure to acidic pH (O'Leary et al., 2015). However, some other researchers reported the up‐regulation of the expression of *Salmonella* virulence genes in neutral pH compared to acidic pH (Roy et al., 2021). Evaluating gene expression in *L. monocytogenes* in the face of the simulated environment of gastric and duodenal aspirate (acidic conditions) showed that the expression of *hly* and *inlC* genes was up‐regulated in almost more than half of the cases; however, *plcA*, *sigB*, *prfA*, *inlB*, *plcB*, *inlA*, *inlP*, *inlJ*, and *lmo2672* gene regulation was not affected (Hadjilouka et al., 2020). The data of another study also showed that the *prfA*, *inlA*, and *hly* genes were significantly induced by HCL. In contrast, the expression of *plcA*, *actA*, *mpl*, and *plcB* genes was not affected after the acid shock (Horlbog et al., 2019). Furthermore, a temperature‐dependent response of *L. monocytogenes* gene expression regulation after an acid shock has been reported so that *prfA*, *inlA*, *inlB*, *inlE*, *bsh*, *clpP*, and the three *usp* gene expression were significantly up‐regulated with environmental temperatures’ decrease (Neuhaus et al., 2013). There is also a report on the up‐regulation of *L. monocytogenes* virulence genes (*hly* and *inlA*) in dried fermented foods under acidic conditions, which occurred despite the stable growth of the bacteria (Martin et al., 2023). As mentioned earlier, this is attributed to the bacterial growth phase; In fact, the bacterial cell in the stationary adapts more to acidic conditions (Faleiro, 2017).

#### 2.1.4 Carbohydrates

According to Delcenserie et al.'s (2012) reports, glucose in low concentrations (0.1%) can significantly down‐regulate the LEE1 operon expression in a luminescent strain of *E. coli* O157:H7. The expression level of *stxA2*, *Ler*, *espA*, and *espD* genes of *E. coli* O157:H7 can be significantly down‐regulated by all concentrations of glucose (0.1, 0.5, and 1%), except for the *stxA2* gene in 1% glucose. However, the *luxS* gene was up‐regulated in all the glucose concentrations, which was noticeable only on 0.1 and 1% glucose, and no difference was observed in the *fliC* gene regulation compared with the control group. Initially, they hypothesized that the down‐regulation in gene expression by glucose was probably due to the suppression of catabolic carbon or a decrease in media pH, which the results showed depended on a factor other than pH because the greatest down‐regulation in gene expression has occurred at the highest pH (Delcenserie et al., 2012). Besides, researchers previously demonstrated that glucose represses the regulation of *L. monocytogenes prfA* virulence gene by entering the cell through the phosphotransferase system and then the phosphorylation of phosphotransferase system (PTS) components (Mertins et al., 2007). Evaluation of the expression of *L. monocytogenes* virulence genes in the presence of some sugars also showed that *prfA* gene expression was affected by none of the sugars (arabitol, arbutin, fructose, maltose, mannose, trehalose, salicin, or cellobiose). However, *hly* gene expression was repressed by the sugars cellobiose and arbutin (Gilbreth et al., 2004). Indeed, *L. monocytogenes* transport carbohydrates (such as cellobiose) by specific phosphoenolpyruvate (PEP)‐dependent phosphotransferase systems and could therefore grow under different environmental conditions (Stoll & Goebel, 2010). It has also been noted that the presence of various carbohydrates (by a signal‐sensing mechanism) in the medium and the utilization of these carbohydrates are very effective in the virulence gene expression of *L. monocytogenes* (Abdelhamed et al., 2020). For the first time, Villoria Recio et al. (2020) reported the antipathogenic properties of the chitin molecule against pathogens, showing that chitin was able to suppress the expression of the *L. monocytogenes* virulence gene (*prfA*) in vitro. Chitin polymer is a complex carbohydrate that the expression of virulence factors down‐regulates by an unknown mechanism's down‐regulation, and unlike other carbohydrates, its function is irrelevant to the phosphotransferase system (Villoria Recio et al., 2020).

#### 2.1.5 Food matrices

Rantsiou, Mataragas, et al. (2012) demonstrated that virulence gene expression of *L. monocytogenes* can be affected by environmental factors in various food matrices. All of the *plcA*, *iap*, *sigB*, and *hly* genes in inoculated *L. monocytogenes* strain on fermented sausage, soft cheese, ultrahigh temperature (UHT) milk, or minced meat were regulated in a strain‐dependent and food‐dependent manner (Rantsiou, Mataragas, et al., 2012). Besides, researchers compared the expression of virulence genes in *L. monocytogenes* grown in minced meat and fermented sausage juices with the level of gene expression in *L. monocytogenes* inoculated into brain heart infusion (BHI) broth and confirmed that gene expression was dependent on the type of food and the strain of *L. monocytogenes*. However, the expression of the virulence gene in different strains of *L. monocytogenes* inoculated into the culture medium regulated similarly in vitro, and it was independent of the strain (Rantsiou, Greppi et al., 2012). Comparison of the virulence gene expression of inoculated *L. monocytogenes* in cantaloupe or BHI broth also showed that transcription of *plcA*, *hly*, *actA*, and *plcB* genes was up‐regulated in inoculated *L. monocytogenes* on cantaloupe, and transcription of *inlA* and *inlB* genes down‐regulated (Kang et al., 2019). While the *hly* and *iap* genes were similarly expressed among various strains of *L. monocytogenes* in laboratory conditions (in BHI broth), significant differences were observed in the gene expression of strains in cheese at 4°C. Moreover, a down‐regulation was noted in the expression of the genes (*plcA*, *hly*, *iap*, and *sigB*) in inoculated *L. monocytogenes* in cheese (at 4 and 12°C) compared with BHI medium at 37°C (Alessandria et al., 2012). Scientists have also reported different levels of induction of *L. monocytogenes* virulence genes in vegetables including cucumber, tomato, and carrot. While the expression of *plcA*, *plcB*, and *inlB* genes in cucumber and that of *sigB*, *prfA*, *hly*, *inlA*, and *inlC* genes in tomato were affected, the gene expression of the bacteria inoculated on carrot's surface did not change compared to the broth. In general, these effects were recorded as a mixed response (up‐ and down‐regulation of genes) that depended on the temperature and storage time of the food matrix (Paramithiotis, Kotsakou, & Drosinos, 2021). Furthermore, most of the virulence genes in *L. monocytogenes* inoculated into ready‐to‐eat salads were expressed in a temperature‐dependent manner, with the highest transcription level reported at 37°C (Marras et al., 2019). Also in *E. coli*, genes were regulated differently in the food matrix compared to the culture medium. Overall, the results of the above studies confirm that interventions in the complex food matrix can control the expression of virulence genes of food pathogens (Fratamico et al., 2011).

#### 2.1.6 Maillard reaction products

The Maillard reaction is a non‐enzymatic reaction that occurs during the thermal processing of foods containing protein and carbohydrates, producing products that cause desirable changes in the food. These compounds also inhibit the growth of microorganisms by chelating metals in a liquid medium (Nooshkam et al., 2020; Yokoyama et al., 2020). In addition, it has been reported that the compounds affect the pathogenicity of some bacteria and down‐regulate virulence gene expression (Diaz et al., 2010). So far, significant inhibition of the growth of various bacteria, such as *L. monocytogenes*, *S. typhimurium*, and *B. cereus*, has been shown by Millard products (Kukuminato et al., 2021; Wong et al., 2023), but little information is available regarding the genotypic effects of these products. Generally, the effect of MRPs can vary depending on the dose of the compounds, the bacterial strain, the type of amino acid available in foods, and other reaction parameters (Kundinger et al., 2008). As reported by Kundinger et al. (2008), the expression of the *Salmonella hilA* gene in a culture medium containing multidrug resistance proteins (MRPs) was significantly up‐regulated, and the lysine MRP had the least effect of inducing the *hilA* gene compared to arginine and histidine (Kundinger et al., 2008). Besides, a 3.43‐fold up‐regulation in stress gene expression has been observed in *Salmonella* exposed to MRP compounds (Chalova et al., 2012). Interestingly, fermented Maillard reaction products (FMRPs) significantly down‐regulated the messenger RNA (mRNA) expression level in Interleukin‐10 (IL‐10), Interleukin‐11 (IL‐11), and Interferon‐γ (IFN‐γ) of *Clostridium perfringens* (Kim et al., 2021). In contrast, there was no change in the expression level of the *cadA* gene of *S. typhimurium* by the media containing MRPs (Diaz et al., 2010). Therefore, these compounds are considered a concern for consumer safety due to the creation of contradictory reactions (induction or suppression of virulence genes of foodborne pathogens) under different conditions (Kundinger et al., 2008).

#### 2.1.7 Redox potential

Redox potential affects physicochemical parameters on microbial growth (Oktyabrskii & Smirnova, 2012). Previously, the role of redox potential in controlling *S. typhimurium* growth and *rpoS* induction has been shown, so artificially down‐regulation of redox potential accelerated *rpoS* induction and growth inhibition of *S. typhimurium*, but in conditions of high redox potential, *rpoS* induction and growth inhibition of *S*. *typhimurium* were suppressed (Komitopoulou et al., 2004). A study by Clair et al. (2010) indicated that most virulence factors produced by *B. cereus* were not mainly regulated in response to redox changes, in fact, the expression pattern of many proteins secreted only differed slightly in response to high and low redox changes (Clair et al., 2010). On the other hand, up‐regulation expression of *nhe* (2.5‐fold) and *hbl* (8‐fold) genes of *B. cereus* has been proven by reducing the redox potential (Ceuppens et al., 2011). These enterotoxins were regulated in response to low redox potential by Fnr (fumarate and nitrate reduction) regulator and the two‐component *resDE* system (Ceuppens et al., 2011; Zigha et al., 2007). According to our research on databases, very little information has been published about the effect of redox potential on the expression of pathogenic genes in food pathogens, and further investigation is needed.

### 2.2 Extrinsic factors

#### 2.2.1 Presence of other microorganisms

Nowadays, the use of bio‐preservatives to maintain food safety and beneficial effects has greatly expanded (Alia et al., 2020; Pakbin et al., 2021; Pilevar & Hosseini, 2013, 2017; Pilevar, Hosseini, Beikzadeh, et al., 2020). It is approved that probiotic bacteria (such as *Lactobacillus* and *Bifidobacterium* strains), as a beneficial group of microbiota for human health, inhibit the growth of foodborne bacteria and affect their virulence potential (Carey et al., 2008). Based on published data, probiotic bacteria such as *Lactobacillus* produce various substances such as lactic acid, bacteriocins, and hydrogen peroxide to inhibit pathogens (Ramezani et al., 2019). However, their mechanism of action on the expression of pathogenic genes is not clearly explained (Carey et al., 2008). Examining the effect of a group of probiotic bacteria (including *Lactobacillus rhamnosus GG*, *Lactobacillus curvatus*, *Lactobacillus plantarum*, *Lactobacillus jensenii*, *Lactobacillus acidophilus*, *Lactobacillus casei*, *Lactobacillus reuteri*, *Pediococcus acidilactici*, *S. cerevisiae*, *Pediococcus pentosaceus*, *Bifidobacterium thermophilum*, *Bifidobacterium boum*, *Brucella suis*, and *Bifidobacterium animalis*) on the expression of the *E. coli* O157:H7 *stx2* gene revealed a different down‐regulation in *stx2* gene expression by each tested probiotic strain. The difference in the rate of down‐regulation in gene expression could be related to the type of acid produced by each strain and the rate of decrease in pH (Carey et al., 2008). In addition, the presence of *Lactiplantibacillus* plantarum B2 alone and combined with the strain *Lactiplantibacillus* spp. B4 decreased the growth of *L. monocytogenes* and down‐regulated the expression of their virulence genes in traditional soft cheese (Martin et al., 2022). It has also been shown that cell‐free spent media (CFSM) of some probiotic strains affect the virulence gene expression of foodborne pathogens. For example, a down‐regulation of virulence genes of *S. typhimurium* (*hilA* and *hilD*) and *E. coli* O157:H7 (*eaeA*) in the presence of *Bifidobacterium bifidum* CFSM bioactive fractions in growth media was reported; *ssrA* and *ssrB* gene expression was also suppressed in *S. typhimurium* plus *B. bifidum* CFSM (Bayoumi & Griffiths, 2012). Moreover, evaluating the effect of *Lactobacilli* cell‐free culture supernatants on *S. aureus* virulence gene expression indicated that the expression of the *sea*, *sae*, *agrA*, *tst*, *spa*, and *spi* genes was significantly down‐regulated in a manner dependent on supernatant's concentration, incubation time, and *Lactobacilli* species, but bacterial growth was not affected by this supernatant (Ramezani et al., 2019). On the contrary, evaluation of the effect of yeast as a biological preservative indicated that the expression of *plcA*, *hly*, and *iap L. monocytogenes* virulence genes in the presence of *Debaryomyces hansenii* in sliced dry‐cured ham was usually up‐regulated to a different rate, which rate was dependent on the gene, the *L. monocytogenes* strain, and water activity (aw) of the dry‐cured ham. Therefore, this yeast is not suggested as a protective culture in meat products (Alia et al., 2020). The researchers also showed a temperature‐dependent virulence gene expression in the *L*. *monocytogenes* co‐cultured in vitro with a bacteriocinogenic *Enterococcus faecium* strain. Generally, a significant down‐regulation was observed at 5°C, but no change occurred at 37°C in most virulence genes (Hadjilouka et al., 2016). Another study reported that the presence of *Saccharomyces cerevisiae* yeast, although not affecting the *L*. *monocytogenes* bacterial population, approximately up‐regulated the expression of their virulence genes in a temperature‐dependent manner (Paramithiotis, Katidi, & Drosinos, 2021). Table 3 illustrates the relative regulation of the virulence genes of pathogens in the presence of other microorganisms.

**TABLE 3:** The relative regulation of the virulence genes of foodborne bacteria in the presence of other microorganisms.

Virulence gene of bacteria		Other microorganisms	Relative fold change			References
*E. coli* O157:H7	*stx2A*	*L. rhamnosus*, *B. thermophilum*, *P. pentosaceus*	−0.40	−0.42	−0.42	Allen and Griffiths (2012)
*C. jejuni*	*ciaB*	*L. acidophilus* La‐5 CFSM, *B. longum* CFSM	−2.8	−5.51		Mundi et al. (2013)
	*flaA*		−7.0	−5.13		
*S. aureus*	*spa*	*Lactobacilli* CFS	−2.18 to −7.98			Ramezani et al. (2019)
	*sea*		−7.89			
	*sae*		−2.18 to −7.98			
	*tst*		−2.18 to −7.98			
	*agr A*		−2.18 to −7.98			
*L. monocytogenes*	*actA*	*B. longum*	−6.2			Tan et al. (2012)
	*hly*		−4.5			
	*inlA*		−6			
	*plcA*		−4.5			

#### 2.2.2 Temperature

Storing food at refrigerated temperature is one of the basic methods that prevent the growth of germs and increase the shelf life of food (Faleiro, 2017). Duodu et al. (2010) showed that virulence genes of *L. monocytogenes* inoculated in salmon at different temperatures (4 and 20°C) were strain‐dependently expressed. No significant differences were reported for *hlyA*, *actA*, *inlA*, and *prfA* gene expression in strain with high virulence potential between incubation at 4 and 20°C. In contrast, the transcription of *inlA* and *hlyA* genes of low virulence strain in salmon at 4°C was significantly lower than their transcription in salmon at 20°C; however, there was no change in *actA* and *prfA* gene transcription in this strain at 4 and 20°C (Duodu et al., 2010). Similarly, an increase in the expression regulation of *Salmonella* virulence genes has been reported at the optimum temperature (37°C) compared to lower temperatures (4, 10, and 25°C). Slama et al. (2013) study showed that the expression levels of *hlyA*, *iap*, *fri*, and *flaA* virulence genes of *L*. *monocytogenes* in incubated cheese at −20°C after 6 months were affected by the freezing factor; they were expressed lower in cold exposure cells than grown cells at 37°C (Slama et al., 2013). In general, many studies have shown that the concomitant use of low storage temperatures with other methods or antimicrobial compounds (such as essential oil, salt, phenolic compounds, or acid) had a more significant effect on the down‐regulation of virulence genes in foodborne pathogens (Bergholz et al., 2012; Carstens, 2017; Neuhaus et al., 2013; Pilevar, Hosseini, Abdollahzadeh, et al., 2020; Rios‐López et al., 2022). In addition, it has been stated that temperature can differently regulate the expression of virulence genes in the biofilm form of *Listeria* bacteria, thereby affecting the pathogenic characteristics. In fact, a temperature‐dependent regulation of gene expression was observed. More precisely, at 20°C, the expression of virulence, stress, and biofilm genes (including *plcA*, *sigB*, *fbpA*, *fbpB*, *lmo1115*, *lmo0880*, and *lmo2089*) was actively down‐regulated (Poimenidou et al., 2023). In this regard, a down‐regulation of the *stx1A* and *stx2A* expression, respectively, −3.35 and −2.9, was reported in the ground beef matrix in the refrigerated storage condition (Mahmoudzadeh, Hosseini, Nasrollahzadeh, et al., 2016).

#### 2.2.3 High‐pressure processing

**FIGURE 3:**
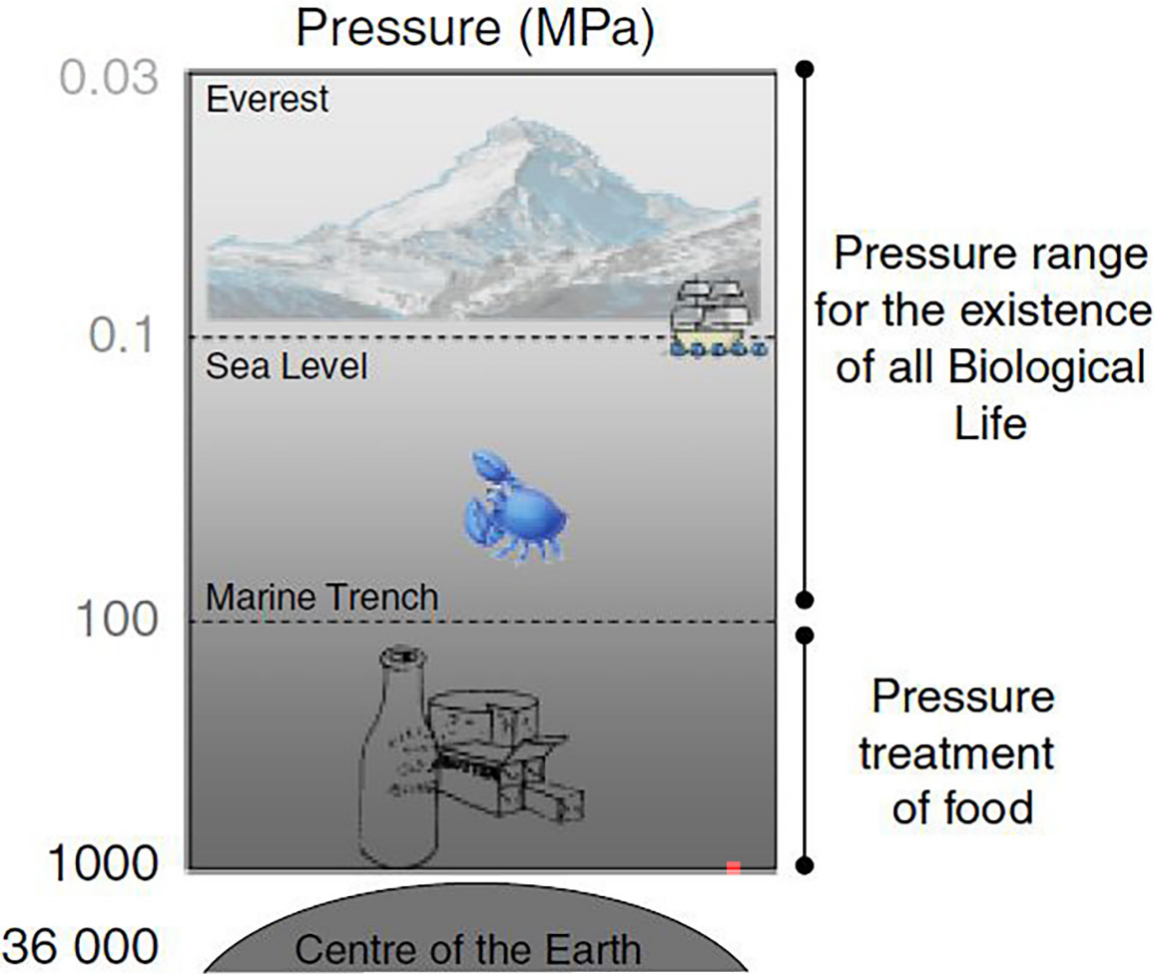
Pressure consumption pattern in food industry (Considine et al., 2008).

The HPP technique is widely used in the food industry to inactivate and eliminate foodborne pathogens, especially in ready‐to‐eat products (Nikparvar et al., 2021). Given that HPP as a novel technology, by applying a pressure of 100 to 1000 MPa (Figure 3), preserves the quality of fresh food and increases the shelf life of products (Considine et al., 2008). Few studies have been conducted on the virulence gene expression of foodborne pathogens in HPP‐treated food. However, there is evidence to show that the virulence gene expression in microorganisms is altered by HPP technology. For example, transcription of *the prfA* virulence gene in *L. monocytogenes* under HPP is highly regulated in vitro (Duru et al., 2021). In Pérez‐Baltar et al. (2021) study, the expression of *L*. *monocytogenes* virulence genes showed a strain‐dependent pattern in dry‐cured ham under HPP. The regulation of *prfA*, *plcA*, *hly*, *sigB*, and *lmo1421* virulence genes was severely down‐regulated in one strain compared in another strain (Pérez‐Baltar et al., 2021). *L*. *monocytogenes* can become more adaptable to HPP and other stressful conditions due to a stress gene (*sigB*) in its gene profile (Wemekamp‐Kamphuis et al., 2004). The *sigB* gene also intensifies the virulence potential of *L*. *monocytogenes* by participating in the regulation of the *prfA* virulence gene (Pérez‐Baltar et al., 2021).

#### 2.2.4 Chemical disinfectants

Despite the widespread use of chemical disinfectants to reduce the microbial load on the surfaces of food processing equipment, some types of foodborne pathogens can survive and enter the food product. On the other hand, multiple changes in gene expression of resistant pathogens have also been reported (Lamas et al., 2019). The results of Romeu et al. (2020) study showed a strain‐dependent up‐regulation in the expression of *S. enteritidis* virulence genes (*invA*, *avrA*, and *csgD*) after treatment with sublethal concentrations of benzalkonium chloride (BAC) and hydrogen peroxide (HP) disinfectants. Moreover, the increased biofilm‐forming ability of strains in the presence of the disinfectants was reported, which could justify the overexpression of virulence genes (Romeu et al., 2020). However, another evaluation had previously shown that the transcriptional level of the *L*. *monocytogenes* virulence genes exposed to benzalkonium chloride did not change (Casey et al., 2014). In another study, down‐regulation of virulence genes of *S*. *typhimurium* was indicated in exposure to chlorhexidine (Condell et al., 2014). However, it was previously reported that hydrogen peroxide disinfectant over‐regulated the *E*. *coli stx2* virulence gene (Allen & Griffiths, 2012). Following hydrogen peroxide treatment of *E*. *coli* serotypes, a down‐regulation of *stx1A*, *stx2A*, and *fliC* virulence gene expression was observed (Mei et al., 2017). The information obtained from these transcripts can provide an accurate understanding of the appropriate treatment by disinfectants so that the new compound directly targets efflux pumps or membrane remodeling systems of microorganisms (Lamas et al., 2019). The relative expression of the virulence genes of pathogens in the presence of some disinfectants is shown in Table 4.

**TABLE 4:** The relative expression of the virulence genes of *E. coli* O157:H7 and *S. enteritidis* in the presence of benzalkonium chloride (BAC) and hydrogen peroxide (HP) disinfectants.

Virulence gene of bacteria		Disinfectants	Relative fold change	References
*E. coli* O157:H7	*stx2A*	HP	+2.1	Allen and Griffiths (2012)
	*stx2B*		+3.4	
*E. coli* O157:H7	*stx1A*		−2.75	Mei et al. (2017)
	*stx2A*		−1.71	
	*fliC*		−1.71	
*S. enteritidis*	*invA*	BAC	>+2	Romeu et al. (2020)
	*avrA*		>+2	
	*csgD*		>+2	

#### 2.2.5 Gaseous atmosphere

So far, many studies have proven the antimicrobial activity of gases such as carbon dioxide (CO2), ozone (O3), and oxygen (O2) at ambient and subambient pressures on important microorganisms in food products (Banerjee et al., 2019; Mortazavi et al., 2021). Interestingly, in the reduced population of *E. coli* by the high dose of 3 μg/g O3, the expression of virulence genes initially down‐regulated and then was significantly regulated with increasing exposure time. However, the expression of *L. monocytogenes* genes down‐regulated even at low doses (1 μg/0.3 g), despite increasing exposure time (Shu et al., 2021). The observation of contradictory responses between *L. monocytogenes* and *E. coli* to O3 can be related to differences in cytoplasmic and membrane structure and components (Shu et al., 2021). In addition, long‐term exposure of *E. coli* to gaseous chlorine dioxide (ClO2), even at low doses (1 μg/g), stimulated gene expression and intensified bacterial defense mechanisms by adapting to the ClO2 in residual populations surviving on the non‐host tomato (Shu et al., 2020). In cucumber packaging with a modified atmosphere, the inoculated *Escherichia coli* population, as well as the expression of motility, adhesion, and oxidative stress genes were inhibited by 2% O2, 7% CO2, and 91% N2 (Sun et al., 2022). Niu et al. (2022) also showed the synergistic effect of high‐pressure CO2 and cinnamon essential oil in inhibiting the expression of *S. typhimurium* virulence genes including *fliC*, *hilA*, *invI*, and *ssrA* (Niu et al., 2022). *CsgD*, *adrA*, *sdiA*, and *luxS* gene expression in *Salmonella enterica* strains was significantly regulated in the presence of micro‐aerobiosis (6.2%–13.2% O2 and 2.5%–9.5% CO2) and anaerobiosis (0.1% O2 and 15% CO2) conditions (Lamas et al., 2016). CO2 reduces the pH of food and controls the growth of microorganisms. This gas has a lethal effect even in low concentrations on forced aerobic organisms (Banerjee et al., 2019; Mortazavi et al., 2021). In general, the results of studies show that in the use of modified atmospheres, gas concentration and exposure time are very important in achieving a safe and desirable result (Shu et al., 2020, 2021).

## 3 CONCLUSION

There are many ambiguities and sometimes contradictions about the function of different antimicrobial agents in regulating the expression of the virulence genes of foodborne pathogens. More studies are needed to elucidate the mechanism of various antimicrobials in suppressing or intensifying the expression of virulence genes. Literature review shows that the virulence genes of foodborne pathogens can be regulated by various intrinsic and extrinsic factors including bacterial strain, type/concentration of antimicrobial compounds, temperature, storage time, food matrix, type/concentration of gaseous atmosphere, redox potential, etc. To achieve a favorable and safe result, all factors affecting the gene expression must be identified and appropriately adjusted. It can be concluded that virulence genes are specifically regulated by external signals; therefore, by controlling the bacterial surroundings environment, the virulence of foodborne pathogens can be reduced.

## AUTHOR CONTRIBUTIONS

**Hedayat Hosseini:** Conceptualization (equal); supervision (equal); validation (equal); writing – original draft (equal). **Razzagh Mahmoudi:** Supervision (equal); validation (equal); writing – original draft (equal). **Babak Pakbin:** Software (equal); writing – review and editing (equal). **Leila Manafi:** Investigation (equal); methodology (equal); project administration (equal); visualization (equal); writing – original draft (equal). **Setayesh Hosseini:** Conceptualization (equal); investigation (equal); project administration (equal); writing – original draft (equal). **Zahra Pilevar:** Methodology (equal); supervision (equal); writing – original draft (equal). **Wolfram Manuel Brück:** Software (equal); writing – review and editing (equal).

## FUNDING INFORMATION

This research is supported by the research grant of Qazvin University of Medical Sciences with the grant number of IR.QUMS.REC.1402.072and is based upon research funded by Iran National Science Foundation (INSF) under project No. 4013451.

## CONFLICT OF INTEREST STATEMENT

The authors declare no conflict of interest.

## Data Availability

The data that support the findings of this study are available on request from the corresponding author.
